# Dual Oxidase-Derived Reactive Oxygen Species Against *Bacillus thuringiensis* and Its Suppression by Eicosanoid Biosynthesis Inhibitors

**DOI:** 10.3389/fmicb.2020.00528

**Published:** 2020-03-27

**Authors:** Seyede Minoo Sajjadian, Yonggyun Kim

**Affiliations:** Department of Plant Medicals, Andong National University, Andong, South Korea

**Keywords:** dual oxidase, reactive oxygen species, gut immunity, benzylideneacetone, *Bacillus thuringiensis*, *Plutella xylostella*

## Abstract

Two entomopathogenic bacteria, *Xenorhabdus* and *Photorhabdus*, are known to be able to synthesize and secrete eicosanoid biosynthesis inhibitors (EIBs) that can enhance pathogenicity of *Bacillus thuringiensis* (Bt) against different target insects. Such enhancements can be explained by the suppression of immune responses in the hemocoel by EIBs. However, little is known about the role of EIBs in the defense against Bt pathogenicity in the gut. This study was focused on the role of insect gut immunity in the defense against Bt pathogenicity, in which the cooperative effect of bacterial metabolites was assessed. Screening 14 different bacterial strains, bacterial culture broth of Photorhabdus *temperata* subsp. *temperata* ANU101 (Ptt) gave the highest cooperative effect on Bt virulence along with significant inhibitory activity against phospholipase A_2_ (PLA_2_) of *Plutella xylostella*. In gut lumen, Ptt culture broth suppressed the generation of reactive oxygen species (ROS) induced by Bt treatment and facilitated bacterial growth, similar to vitamin E, an antioxidant. To analyze the ROS source, dual oxidase (*Px-Duox*) and NADPH-dependent oxidase (*Px-Nox*) genes were predicted from *P. xylostella* genome and their expressions were confirmed in larval gut. RNA interference (RNAi) of *Px-Duox* expression reduced ROS levels in both gut epithelium and lumen while RNAi of *Px-Nox* expression reduced ROS levels only in gut epithelium. Ptt extract significantly suppressed gene expression levels of *Px-Duox* and *Px-Nox*, leading to lower ROS concentrations in the gut lumen. Three commercial PLA_2_ inhibitors significantly increased the insecticidal activity of Bt by suppressing ROS levels in the gut lumen. These results indicate that Ptt extract containing EBIs can prevent up-regulation of ROS level in the midgut in response to Bt infection and enhance the virulence of Bt against *P*. *xylostella*.

## Introduction

*Bacillus thuringiensis* (Bt) is a Gram-positive, rod-shaped, and endospore-forming bacterium with entomopathogenic property (Raymond et al., 2009). Bt crystalline (Cry) protoxins are solubilized in insect gut lumen under alkaline pH and activated to bind to gut epithelial membrane (Bietlot et al., 1989). Cry toxin bound to epithelial membrane can make membrane pores and induce cell lysis, which allows bacteria to pass from gut lumen to hemocoel and finally induce a fatal septicemia (Broderick et al., 2006). Alternatively, the bound Cry toxin can trigger epithelial cell death via intracellular cAMP signal (Zhang et al., 2016). Along with their high insecticidal activity and relative safety to mammals and livestock, Bt and its toxins have been used as efficient biocontrol agents against agricultural and medical insect pests (Bravo et al., 2011).

Like other organisms, all insects are exposed to different types of microorganisms during their life. Once entering the body cavity through wounds or other pathogens’ infection, microbes can be recognized by host insects to induce cellular and humoral immune responses (Lemaitre and Hoffmann, 2007). Cellular immune responses include hemocyte-mediated phagocytosis, encapsulation, and nodule formation via activating hemocyte-spreading behavior (Ahmed and Kim, 2019). Humoral immune responses are executed by various antimicrobial peptides (AMPs) and activated at relatively later stage of infection to remove the remaining microbes after activating cellular immunity (Haine et al., 2008; Kim et al., 2018a).

Gut epithelium is constantly exposed to non-pathogenic and pathogenic food-borne microbes that can activate gut immunity to protect insects (Engel and Moran, 2013; Lemaitre and Miguel-Aliaga, 2013). Some of these bacterial microorganisms can permanently colonize gut lumen with various symbiotic relationships, including toxin and other xenobiotic compound decomposition (Engel and Moran, 2013), host development (Shin et al., 2011), food digestion and energy extraction (Tremaroli and Bäckhed, 2012), and development of immune system (Weiss et al., 2011; Broderick et al., 2014). The following two types of molecular effectors are important in the regulation of gut immune response: (1) reactive oxygen species (ROS) produced by dual oxidase (Duox) or NADPH-dependent oxidase (Nox) and (2) AMPs produced by gut epithelium via immune deficiency (IMD)/nuclear factor-κB (NF-κB) signaling pathways (Ryu et al., 2006; Buchon et al., 2013). These two effectors can act synergistically to reduce the growth and proliferation of invading microorganisms (Yao et al., 2015). All bacteria including symbiotic and pathogenic bacteria can produce pathogen-associated molecular patterns such as peptidoglycan that can induce gut immune response through IMD pathway (Zaidman-Rémy et al., 2006). In the absence of gut infection, the presence of a commensal-derived peptidoglycan can constitutively activate this pathway at a low level by various negative regulators (Zaidman-Rémy et al., 2006; Kleino et al., 2008; Maillet et al., 2008). However, acute pathogenic gut infection can result in the release of large quantities of peptidoglycan fragments (Buchon et al., 2013) known to induce the IMD pathway to trigger AMP production. Moreover, homeobox protein, Caudal, can selectively repress IMD/NF-kB-dependent AMPs to protect symbiotic bacteria and host health (Ryu et al., 2008). In contrast with IMD pathway activation, *Duox* expression is activated by pathogens, but not by symbionts (Ha et al., 2009a; Bae et al., 2010; Lee et al., 2013). Consequently, gut immunity is critical and important for normal development by fostering symbiotic microorganisms and for survival upon infection by defeating pathogens (Lemaitre and Hoffmann, 2007; Liu et al., 2013).

Gut immunity has been extensively evaluated through an oral infection in *Drosophila melanogaster* as a model insect (Ha et al., 2005; Ryu et al., 2006; Zaidman-Rémy et al., 2006; Nehme et al., 2007). Several studies have also shown that in some lepidopteran insects, ingested microorganisms can induce immunity responses in the hemocoel. In *Galleria mellonella*, larvae fed with non-pathogenic and pathogenic bacteria show increased lysozyme and phenoloxidase activity in the hemolymph with induction of some AMPs (Freitak et al., 2014). In another study, when *Bombyx mori* larvae were fed with *Bacillus bombysepticus*, cellular and humoral immunities in the host insect were activated along with activation of endocrine and metabolism genes (Huang et al., 2009). Thus, oral infection can induce immune responses in both gut lumen and the hemocoel.

*Xenorhabdus* and *Photorhabdus* are two groups of entomopathogenic bacteria that can suppress immune responses of target insects including lepidopteran species by synthesizing and releasing secondary metabolites (Forst et al., 1997; Casanova-Torres et al., 2017). With the help of host nematodes (*Steinernema* or *Heterorhabditis*), bacteria are released into insect hemocoel, in which these bacteria can suppress insect immune responses by inhibiting enzyme activity of phospholipase A_2_ (PLA_2_), subsequently shutting down the production of eicosanoids (Kim et al., 2005). Eicosanoids are a group of oxygenated C20 polyunsaturated fatty acids that can mediate cellular and humoral immune responses in insects, such as hemocyte-spreading behavior which is involved in phagocytosis, nodulation, and encapsulation (Stanley and Kim, 2019). However, the role of eicosanoids in mediating gut immunity in insects remains unknown.

*Xenorhabdus* and *Photorhabdus* have been predicted to produce various secondary metabolites based on their biosynthetic gene clusters such as genes involved in non-ribosomal peptide synthetase and polyketide synthase (Tobias et al., 2017) to alter target insect physiological processes. Especially, it would be favorable for *Xenorhabdus* or *Photorhabdus* to inhibit eicosanoid biosynthesis of target insects to avoid fatal immune attacks. Indeed, the bacteria synthesize and release PLA_2_ inhibitors to induce immunosuppression in insect hemocoel (Seo et al., 2012). Furthermore, the bacterial culture broths containing these secondary metabolites are used to enhance insecticidal activities of Bt and other entomopathogens, which exhibit virulence activity in insect midgut (Jung and Kim, 2007; Kwon and Kim, 2008). However, it was not clearly understood how *Xenorhabdus* or *Photorhabdus* metabolites enhance the Bt pathogenicity.

The aim of this study was to explain the cooperative effect of *Xenorhabdus*/*Photorhabdus* bacterial metabolites on Bt pathogenicity. To address this issue, effects of bacterial metabolites on gut immunity were assessed by quantifying Duox/Nox-derived ROS. First, this study tested the immunological role of ROS in the defense against Bt pathogenicity. Second, *Duox*/*Nox* genes were identified from *Plutella xylostella* and their roles in producing ROS were analyzed. Third, effects of bacterial metabolites in suppressing ROS generation by inhibiting *Duox*/*Nox* expression were determined. Finally, this study validated the PLA_2_-inhibitory activity of bacterial metabolites in suppressing ROS levels in gut lumen and enhancing Bt toxicity with known PLA_2_ inhibitors.

## Materials and Methods

### Insect Rearing and Bacterial Culturing

*Plutella xylostella* larvae were reared with cabbage leaves at 25 ± 1°C with a 16:8 h (L:D) photoperiod. Under these rearing conditions, *P. xylostella* underwent four larval instars (“L1–L4”) before pupation. Adults were fed with 10% sucrose for oviposition.

*Bacillus thuringiensis* subsp. *kurstaki* (“BtK”) was obtained from Hanearl Science (Taebaek, Korea). It was cultured in a nutrient broth medium (0.5% peptone and 0.3% beef extract) at 30°C for 48 h. These cultured bacteria were then further incubated at 4°C for at least 24 h to allow bacteria to form endospores. Fourteen strains of *Xenorhabdus* and *Photorhabdus* were obtained from previous collections [*Xenorhabdus nematophila* K1 (“XnK1”) (Park et al., 1999), *Xenorhabdus hominickii* ANU101 (“Xh”) (Park et al., 2017), *Xenorhabdus ehlersii* KSY (“Xe”) (Kim et al., 2018b), *Photorhabdus temperata* ssp. *temperata* ANU101 (“Ptt”) (Kang et al., 2004), *X. nematophila* GNUS-143 (“XnG143”), and *X. hominickii* DY1 (“XhDY”) (Hasan et al., 2019)] and from Korean Agricultural Culture Collection (KACC, RDA, Jeonju, Korea) [*Photorhabdus luminescens* KACC12123 (“Pl”), *P. luminescens* subsp. *laumondii* KACC12283 (“Pll”), *P. luminescens* subsp. *thracensis* KACC12284 (“Plt”), *X. nematophila* KACC12145 (“Xn12154”), *X. nematophila* Mexico (“Xnmex”), *X. nematophila* France (“XnF”), *Xenorhabdus bovienii* (“Xb”), and *Xenorhabdus poinarii* (“Xp”)]. These bacteria were cultured in Luria–Bertani (LB) medium at 28°C for 48 h.

### Chemicals

Benzylideneacetone (BZA), 4-bromophenacyl bromide (BPB), and methyl arachidonyl fluorophosphonate (MAFP) were purchased from Sigma–Aldrich Korea (Seoul, South Korea). Bromoenol lactone (BEL) was purchased from Cayman Chemical (Ann Arbor, MI, United States). All inhibitors were dissolved in dimethyl sulfoxide (DMSO). Vitamin E (α-tocopherol, Sigma–Aldrich Korea) was also dissolved in DMSO.

### Preparation of Bacterial Culture Broth

To obtain bacterial culture broths, different strains of *Photorhabdus* or *Xenorhabdus* bacteria were cultured in 100 mL of LB for 48 h at 28°C until OD600 reached 2.0. The culture medium was then centrifuged at 8000 r/min for 20 min. The supernatant was collected and filtered through 0.22 μm syringe filter (Pall Corp., Ann Arbor, MI, United States) to remove bacterial cells.

### Insecticidal Bioassay and Median Lethal Parameters

Btk mixture was prepared by dissolving BtK cells in 10 mL of the filtered bacterial extract of *Xenorhabdus* or *Photorhabdus* culture broth. Control was a mixture of BtK and uncultured LB medium. Bioassay used leaf-dipping method by soaking cabbage leaves in different mixtures for 10 min and subsequent drying under darkness for 30 min before applying to test larvae (1 day old L4). Each treatment was replicated three times. Each replication used 10 larvae of *P. xylostella*. To measure median lethal time (LT_50_), BtK in the bacterial culture broth was prepared at 1.5 × 10^6^ spores/mL. The number of dead individuals was counted for 4 days. To calculate the median lethal concentration (LC_50_), five different concentrations (0, 5 × 10^5^, 1 × 10^6^, 2 × 10^6^, 4 × 10^6^ spores/mL) of BtK were prepared in each bacterial extract. LC_50_ was measured after 48 h post treatment based on the number of the dead individuals. PoloPlus version 2.0 (LeOra software 2005, Berkeley, CA, United States) was used for Probit analysis to calculate LT_50_ and LC_50_.

### Bioinformatics—Gene Acquisition and Domain Analysis

Sequences of *P. xylostella* Duox (*Px-Duox*) and Nox (*Px-Nox*) were obtained from GenBank^1^ with accession numbers of XP_011558844.1 and XP_011562708.1, respectively. Their open reading frame (ORF) sequences were deposited at GenBank with accession numbers of XP_011558844.1 and XP_011562708.1, respectively. Protein domain structure was predicted using HMMER^2^ and Pfam^3^. Phylogenetic analyses were performed using the neighbor-joining method with MEGA6 and ClustalW programs. Bootstrapping values were obtained with 1000 repetitions to support branching and clustering.

### RNA Extraction and RT-qPCR

Total RNAs were extracted from treated larvae using Trizol reagent (Invitrogen, Carlsbad, CA, United States) according to the manufacturer’s instruction. Extracted RNAs were dissolved in 50 μL of diethyl pyrocarbonate-treated deionized and distilled water. Their concentrations were quantified with a spectrophotometer (Nanodrop, Thermo Scientific, Wilmington, DE, United States). First strand cDNAs were synthesized using 1 μg of total RNA and Maxime RT PreMix (Intron Biotechnology, Seoul, South Korea) containing oligo dT primer according to the manufacturer’s instruction. Synthesized cDNAs were used to construct double-stranded RNA (dsRNA) or as templates for qPCR amplification. qPCR was conducted using 2X SYBR Green Real-time PCR Master Mix (Toyobo, Osaka, Japan) including 5 mM of gene-specific forward and reverse primers using 80 ng of cDNA as template in a final reaction volume of 20 μL. Temperature cycles used for qPCR were: 95°C for 10 min for initial heat treatment followed by 40 cycles of 98°C for 20 s, 52°C for 30 s, and 72°C for 30 s. A cytoskeletal gene, β-actin, was assessed along with test samples as an endogenous control. Melting curves of products were obtained to confirm amplification specificity. Quantitative analysis was done with a comparative C_*T*_ method using the reference gene (Livak and Schmittgen, 2001) to estimate mRNA expression levels. The experiment was independently replicated three times. All gene-specific primers and their sequences are described in Supplementary Table S1.

### Analysis of AMP Gene Expression

After Bt or Ptt treatment, expression levels of downstream immune associated genes were detected by RT-qPCR with gene specific primers of seven AMP genes (Supplementary Table S2). Total RNAs were extracted from treated samples. First strand cDNAs were synthesized for RT-qPCR. β*-Actin* was amplified as the housekeeping gene for internal standardization of RT-qPCR assay. All samples were analyzed in triplicate during RT-qPCR analysis. Data obtained from RT-qPCR experiments were expressed as means ± SE.

### Construction of Double-Stranded RNA (dsRNA) and RNA Interference (RNAi)

RNA interference (RNAi) was performed using gene-specific dsRNA which was prepared using a MEGAscript RNAi kit (Ambion, Austin, TX, United States) according to the manufacturer’s instruction. *Px-Duox* and *Px-Nox* DNA fragments were obtained by PCR using gene-specific primers (Supplementary Table S1) containing T7 promoter sequence at the 5′ end. Sense and antisense RNA strands were synthesized using T7 RNA polymerase at 37°C for 4 h. The resulting dsRNA was mixed with transfection reagent Metafectene PRO (Biontex, Plannegg, Germany) at 1:1 (v/v) ratio and then incubated at 25°C for 30 min to form liposomes; 1 μg of dsRNA was injected to larval hemocoel using a microsyringe (Hamilton, Reno, NV, United States) equipped with a 26-gauge needle. At 24, 48, and 72 h post-injection (PI), RNAi efficacy was determined by RT-qPCR as described above. Each treatment was replicated three times. At 48 h PI, treated larvae were used to measure intracellular and extracellular ROS levels after an oral infection.

### ROS Quantification

Reactive oxygen species was quantified using an OxiSelect Intracellular ROS Assay Kit (Cat. No. STA-342, Cell Biolabs Inc., San Diego, CA, United States). Briefly, dissected guts were centrifuged at 1000 r/min for 2 min to separate its content and tissue. Gut cells were washed twice with 1 mL of 100 mM phosphate-buffered saline (PBS, pH 7.4) and centrifuged at 1000 r/min for 5 min at room temperature. These cells were then resuspended in TC-100 cell culture medium containing 0.1 × DCFH-DA (dichlorofluorescein diacetate, 20 × DCFH-DA stock, Part No. 234201) and incubated at 37°C for 30 min with gentle mix by inverting the tubes. Cells were then washed three times in PBS and dissolved in TC-100 cell culture media. After adding 250 μL of 2X Cell Lysis Buffer (Part No. 234203) to the same volume of this suspension (250 μL), the mixture was incubated at room temperature for 5 min. Each 150 μL of cell lysate was transferred to a 96-well plate. Fluorescence was then read at 530 nm emission after 480 nm excitation. For extracellular ROS measurement, DCFH-DA was mixed with gut content, incubated at 37°C for 30 min, and shaken gently by inverting the tubes. To validate the fluorescence value, calibration curve was drawn using a serial dilution of dichlorofluorescein (DCF) standard (Part No. 234202) in TC-100 cell culture media.

### PLA_2_ Activity Measurement

Secretory PLA_2_ (sPLA_2_) activity in larval hemolymph and fat body of *P. xylostella* was fluorometrically determined using a commercial assay kit (sPLA_2_ Assay Kit, Cayman Chemical, Ann Arbor, MI, United States) with diheptanoyl thio-PC as enzyme substrate was used using the method described by Vatanparast et al. (2018). Cellular PLA_2_ (cPLA_2_) activity measurement used the same kit but used different substrate, arachidonyl thio-PC. A spectrofluorometer (VICTOR multi-label Plate reader, PerkinElmer, Waltham, MA, United States) was used to measure enzyme activity. Each treatment was replicated with three biologically independent enzyme preparations using different larval samples. Specific enzyme activity (μmol/min/mg) was calculated by dividing absorbance change by protein amount used as enzyme source for the reaction. Protein amount was measured by Bradford (1976) method.

### Statistical Analysis

All bioassays were conducted in three independent biological replicates and plotted by mean ± standard error using Sigma plot. Means and variances of treatments were compared by a least squared difference (LSD) test of one-way analysis of variance (ANOVA) using PROC GLM of SAS program (SAS Institute, Inc.) and discriminated at Type I error = 0.05.

## Results

### Culture Broths of *Xenorhabdus* and *Photorhabdus* Enhance BtK Pathogenicity

**FIGURE 1:**
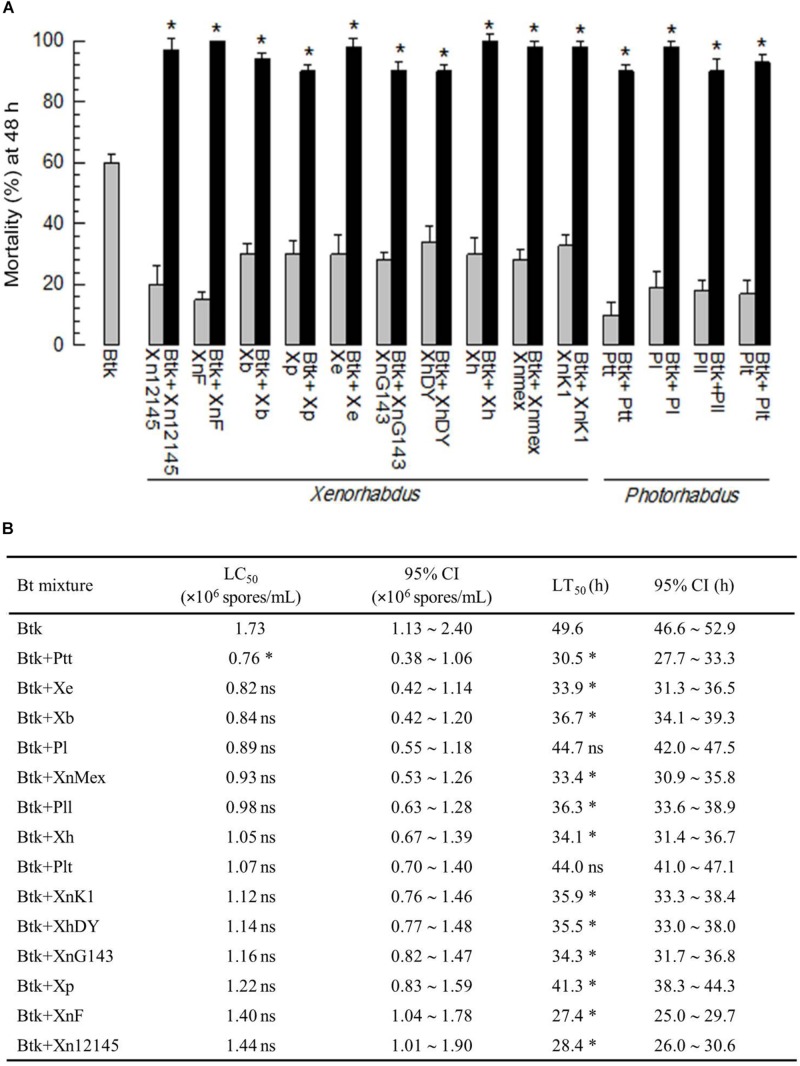
Enhanced Bt toxicity by culture broth of *Xenorhabdus* or *Photorhabdus* against *P. xylostella* larvae. **(A)** Effects of 14 bacterial species culture broth on pathogenicity of *B. thuringiensis kurstaki* (BtK) against one day old L4 larvae of *P. xylostella*. The mixture was prepared by suspending BtK cells (1.5 × 10^6^ spores/mL) in 100 mL of the bacterial cultured broth which was obtained when OD_600_ reached to 2.0. Control (BtK only) was prepared by suspending BtK cells in uncultured medium. Leaf-dipping bioassay was performed as described in Materials and methods. Each treatment used 10 larvae and replicated three times. Mortality was measured at 48 h after treatment. Asterisk indicates significant difference between control (BtK only) and treatment (BtK + bacterial culture broth) at Type I error = 0.05 (LSD test). **(B)** Median lethal concentration (LC_50_) of BtK under mixture with different bacterial culture broths. In this assay, five different concentrations (0, 5 × 10^5^, 1 × 10^6^, 2 × 10^6^, 4 × 10^6^ spores/mL) of BtK were prepared for each bacterial extract. LC_50_ was measured at 48 h after treatment based on the number of dead individuals. To measure median lethal time (LT_50_), BtK in the bacterial culture broth was prepared at 1.5 × 10^6^ spores/mL. The number of dead individuals was counted for four days. Each treatment consisted of three independent replications with 10 larvae. Asterisk represents non-overlapping in 95% confidence interval (CI) compared to that of BtK alone while “ns” represents overlapping of 95% CI. All bacterial acronyms are explained in Section “Materials and Methods.”

A low dose (1.5 × 10^6^ spores/mL) of BtK resulted in only about 60% mortality against *P. xylostella* L4 larvae by leaf-dipping bioassay (Figure 1A). However, an addition of *Xenorhabdus* or *Photorhabdus* bacterial culture broth to BtK treatment significantly (*P* < 0.05) increased the pathogenicity. In contrast, the culture broth alone did not give high mortality to the larvae. When cooperative effects of bacterial culture broths of the 14 bacterial strains were compared, the culture broth from *P. temperata temperata* (Ptt) was the most potent one in decreasing median lethal concentrations of BtK and increasing its speed-to-kill (Figure 1B). Thus, it was selected for further studies.

### Ptt Culture Broth Suppresses PLA_2_ Activities in Hemolymph and Fat Body

**FIGURE 2:**
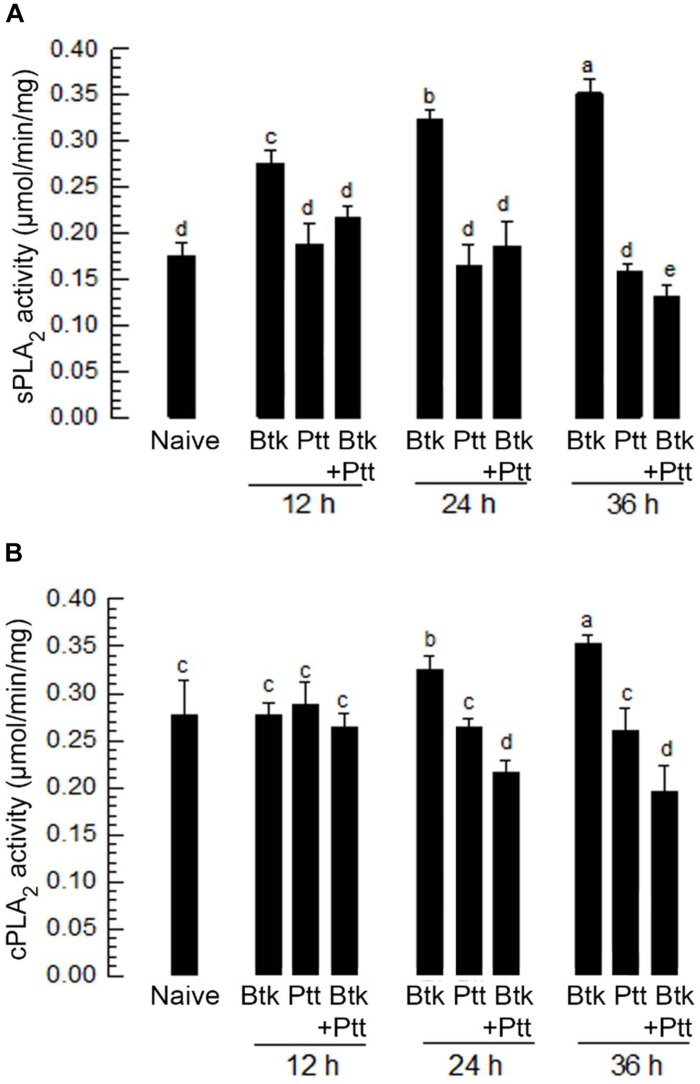
Influence of *B. thuringiensis kurstaki* (BtK) treatment on PLA_2_ activity of *P. xylostella* and its suppression by *P. temperata temperata* (Ptt) culture broth. **(A)** sPLA_2_ activity in plasma and **(B)** cPLA_2_ activity in fat body of *P. xylostella* larvae. Bacterial mixture treatment was prepared by suspending BtK cells (1.5 × 10^6^ spores/mL) in 100 mL of the bacterial cultured broth which was obtained when OD_600_ reached to 2.0. BtK only was prepared by suspending BtK cells in uncultured medium. Ptt only represented the bacterial cultured broth. Leaf-dipping bioassay was performed to apply treatments as described in Section “Materials and Methods.” Each treatment was replicated three times. Each replication used 10 L4 larvae of *P. xylostella*. Different letters indicate significant differences among means at Type I error = 0.05 (LSD test).

Upon BtK oral treatment, PLA_2_ activities in hemolymph and fat body were assessed (Figure 2). Hemolymph showed relatively high level of sPLA_2_ activity without BtK treatment (Figure 2A). The basal level of sPLA_2_ activity was significantly (*P* < 0.05) enhanced by BtK treatment for at least 36 h. In contrast, oral administration of Ptt culture broth did not change the sPLA_2_ activity in hemolymph. The addition of Ptt culture broth to BtK treatment significantly (*P* < 0.05) prevented the induction of sPLA_2_ activity. Similarly, cPLA_2_ activity was enhanced by BtK oral treatment with a slight delay (Figure 2B). The addition of Ptt culture broth also inhibited the up-regulation of cPLA_2_ activity induced by BtK treatment. These results supported results of a previous report (Seo et al., 2012) showing that the Ptt culture broth contained PLA_2_ inhibitor(s) to inhibit eicosanoid biosynthesis. This result also suggests that the PLA_2_-inhibitory factor(s) is delivered from the gut lumen to hemocoel. Here, we needed to assess the effect of PLA_2_-inhibitory factor(s) on gut immunity.

### Ptt Culture Broth Suppresses ROS in Gut Lumen and Epithelium

**FIGURE 3:**
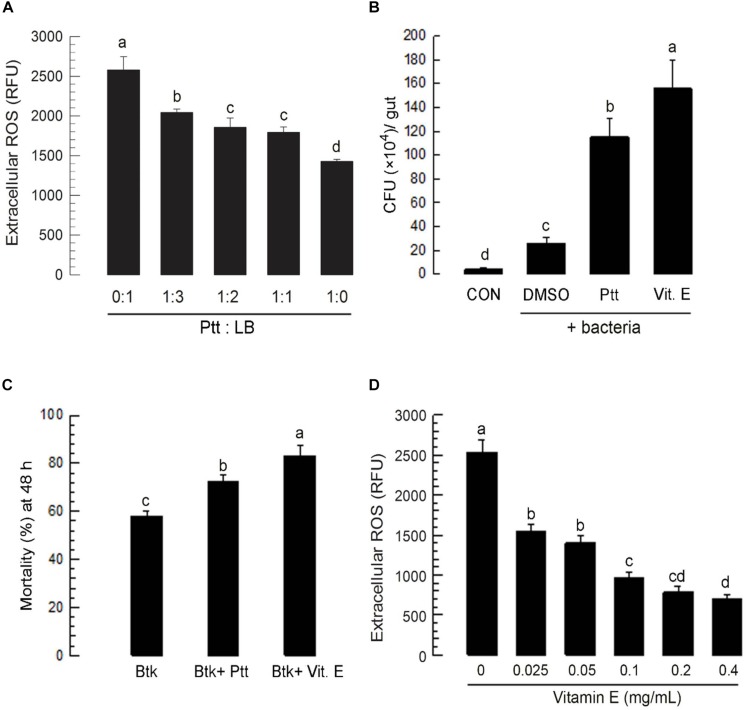
Anti-oxidant activity of *P. temperata temperata* culture broth (“Ptt”) against ROS in the gut lumen of L4 larvae of *P. xylostella*. Ptt represents the bacterial cultured broth which was obtained when OD_600_ reached to 2.0. **(A)** Dose effects of Ptt on ROS levels in the gut lumen. Ptt was diluted with fresh LB medium in different ratios to obtain different concentrations. *P. xylostella* larvae were fed with cabbage leaves treated with different concentrations of Ptt. After 24 h, gut lumen contents were isolated and used to measure ROS levels. Each treatment was replicated three times. Each replication used 10 larvae. **(B)** Suppression of antibacterial activity of gut lumen by Ptt treatment. Ptt bacteria were treated along with Ptt culture broth or vitamin E (“Vit. E,” 0.1 mg/mL) by leaf-dipping method. At 6 h post feeding, gut lumen contents were isolated and plated onto LB medium to count Ptt colonies (red-colored) to obtain colony-forming unit (CFU)/gut. Each treatment was replicated three times. Each replication used 10 larvae. **(C)** Influence of anti-oxidants (Ptt and Vit. E) on pathogenicity of *B. thuringiensis kurstaki* (BtK) against *P. xylostella* larvae. Bacterial mixture treatment (BtK + Ptt) was prepared by suspending BtK cells (final concentration: 1.5 × 10^6^ spores/mL) in 1:1 diluted Ptt medium. BtK only was prepared by suspending BtK cells in uncultured medium. “BtK + Vit. E” was prepared by suspending BtK cells (final concentration: 1.5 × 10^6^ spores/mL) in 100 mL of uncultured LB medium containing Vit. E at a final concentration of 0.1 mg/mL. Leaf-dipping bioassay was performed to apply treatments as described in Section “Materials and Methods.” Each treatment was replicated three times. Each replication used 10 L4 larvae of *P. xylostella*. **(D)** Dose response of Vit. E on ROS levels in the gut lumen. Vit. E was diluted with distilled water to obtain different concentrations. *P. xylostella* larvae were fed with cabbage leaves treated with different concentrations of Ptt. After 24 h, gut lumen contents were isolated and used to measure ROS levels. Each treatment was replicated three times. Each replication used 10 larvae. Different letters indicate significant differences among means at Type I error = 0.05 (LSD test).

To investigate the role of Ptt culture broth in enhancing BtK pathogenicity in the gut of *P. xylostella* larvae, its anti-oxidant activity was assessed by monitoring changes of ROS levels after its oral administration (Figure 3A). Ptt culture broth significantly (*P* < 0.05) suppressed ROS levels in the gut lumen in a dose-dependent manner. The decrease of ROS level in the gut lumen induced by Ptt culture broth was favorable for bacterial growth. Similar increase of bacterial growth was achieved by the addition of vitamin E, an anti-oxidant (Figure 3B). Vitamin E treatment increased BtK pathogenicity (Figure 3C) by suppressing ROS level in the gut lumen (Figure 3D).

### Identification of Nox and Duox Genes From *P. xylostella*

**FIGURE 4:**
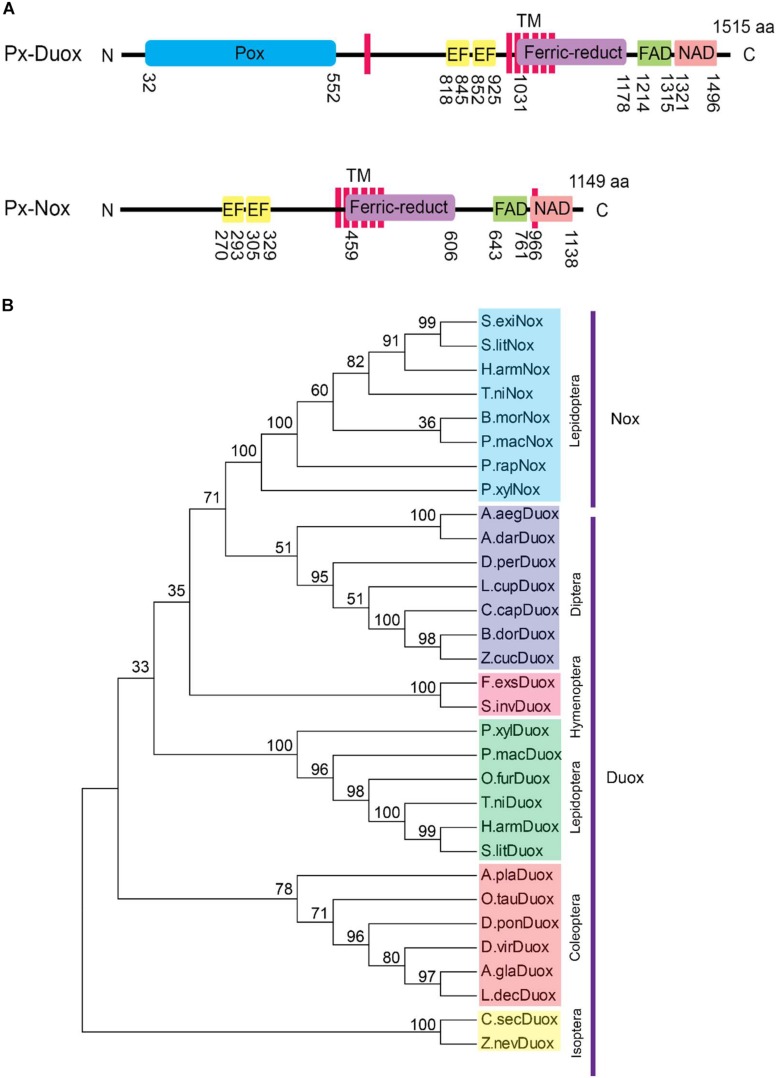
Prediction of dual oxidase (Px-Duox) and NADPH-dependent oxidase (Px-Nox) of *P. xylostella*. **(A)** Functional domain analysis of Px-Duox (1515 amino acids) and Px-Nox (1149 amino acids). Each domain is denoted by positions of amino acid residues. Predicted domains include “Pox” for peroxidase, “EF” for calcium-binding EF hand, “TM” for transmembrane, “Ferric-reduct” for ferric chelate reductase, “FAD” for FAD-binding domain, and “NAD” for NAD-binding domain. **(B)** A phylogenetic analysis of Px-Duox and Px-Nox with other insect Duox and Nox genes based on their amino acid sequences. The analysis was performed using MEGA6. Bootstrap numbers were obtained by 1000 repetitions. GenBank accession numbers are displayed in Supplementary Table S3.

Interrogation to the genome of *P. xylostella* allowed us to predict two ROS production-associated oxidase genes, *Px-Nox* and *Px-Duox* (Figure 4). Two oxidase genes shared most of functional domains except N-terminal peroxidase in *Px-Duox* (Figure 4). Phylogenetic analysis indicated that Px-Duox and Px-Nox were distinctly clustered (Figure 4B). In each cluster, they showed closeness with other lepidopteran orthologs.

**FIGURE 5:**
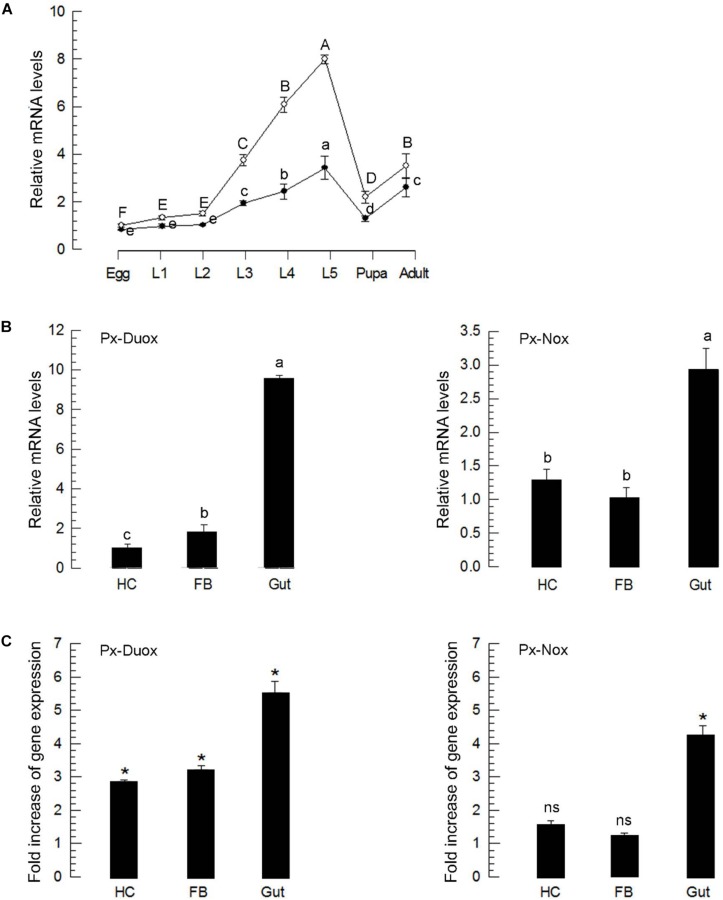
Expression profiles of *Px-Duox* and *Px-Nox*. **(A)** Expression patterns in different developmental stages: egg, first to fourth instar larvae (“L1–L4”), pupa, and adult. **(B)** Expression patterns in different tissues of L4 larvae: hemocyte (“HC”), fat body (“FB”), and gut (“Gut”). Different letters indicate significant differences among means at Type I error = 0.05 (LSD test). **(C)** Induction of *Px-Duox* and *Px-Nox* expressions in response to bacterial challenge of *Bacillus thuringiensis kurstaki* (BtK). L4 larva were fed with BtK-treated cabbage leaves for 12 h. BtK suspension was prepared at a concentration of 1.5 × 10^6^ spores/mL. Expression levels in different tissues of treated L4 were expressed as fold changes compared to expression levels in naive larvae. Asterisks indicate significant differences between naïve and bacterial challenge at Type I error = 0.05 (LSD test). “ns” represents no significant difference. All these expression experiments were replicated with three independent tissue preparations. Each replication used 15 insects. A cytoskeletal gene, β*-actin*, was used as a reference to normalize expression level.

Both oxidases were expressed in all developmental stages (Figure 5A). In all developmental stages, *Px-Duox* was expressed at higher levels than *Px-Nox*. Especially, *Px-Duox* was highly expressed in the gut (Figure 5B). In response to BtK treatment, *Px-Duox* expression was significantly (*P* < 0.05) up-regulated in all three tissues (hemocytes, fat body, and gut) whereas *Px-Nox* expression was up-regulated only in the gut (Figure 5C). Both oxidase genes were highly up-regulated in the gut in response to BtK infection.

### RNAi of *Px-Duox* or *Px-Nox* Expression Suppresses ROS Level in Gut and Increases BtK Pathogenicity

**FIGURE 6:**
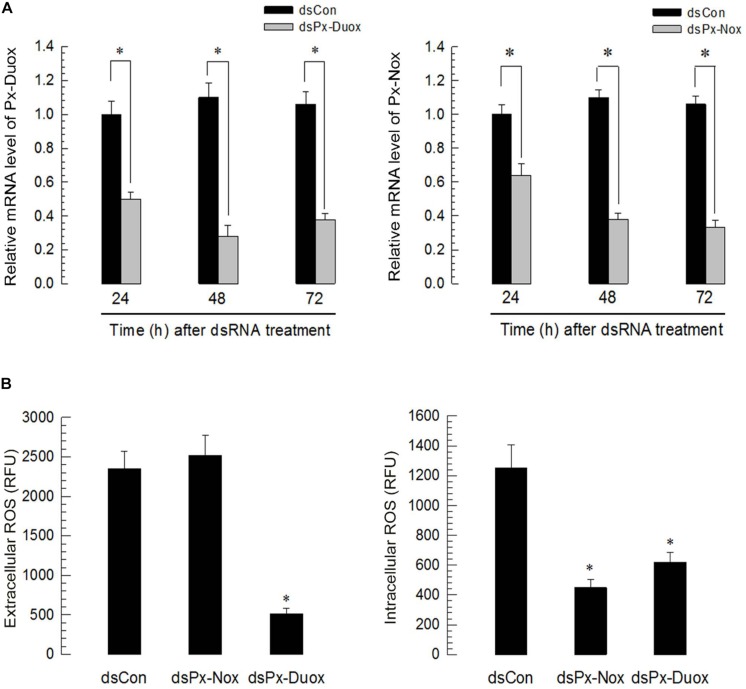
RNA interference (RNAi) of *Px-Duox* and *Px-Nox* expressions and subsequent down-regulation of ROS levels in the gut of *P. xylostella* L4 larvae. **(A)** Temporal RNAi patterns after injection of gene-specific double-stranded RNA (dsRNA, 1 μg/larva) in the larval gut. A viral gene, *CpBV302*, was used as a control dsRNA (“dsCon”). A cytoskeletal gene, β*-actin*, was used as reference expression level in each test sample. Asterisks indicate significant differences between control and treatment at each time point (LSD test at Type I error = 0.05). All these expression analyses were replicated with three independent tissue preparations. Each replication used 15 insects. **(B)** Effect of Px-Duox and Px-Nox RNAi on ROS levels in gut lumen (“extracellular”) and gut epithelium (“intracellular”). To induce ROS levels, BtK (1.5 × 10^6^ spores/mL)-treated cabbage leaves were fed to larvae at 48 h post dsRNA treatment. At 12 h post bacterial treatment, intestinal ROS levels were measured. Each treatment was replicated three times and each replication used 15 larvae. Asterisk indicates significant difference between control and treatment at Type I error = 0.05 (LSD test).

Silencing of *Px-Duox* or *Px-Nox* expression was performed by hemocoelic injection of gene-specific dsRNA to *P. xylostella* larvae (Figure 6). RNAi efficiencies were assessed by qPCR and showed that RNAi condition was maintained for at least 72 h PI in two oxidase genes (Figure 6A). RNAi specific to *Px-Duox* suppressed ROS levels in both gut lumen and epithelium (Figure 6B). However, RNAi of *Px-Nox* expression suppressed ROS levels in the gut epithelium, but not in the gut lumen.

**FIGURE 7:**
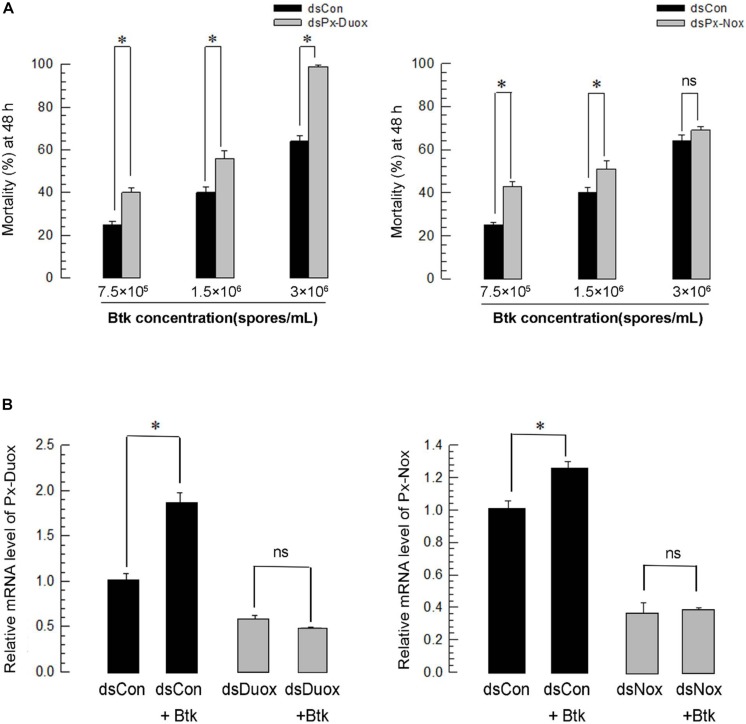
Effect of *Px-Duox* or *Px-Nox* RNA interference (RNAi) on susceptibility of *P. xylostella* to entomopathogen. RNAi was performed by injecting gene-specific double-stranded RNA (dsRNA, 1 μg/larva). A viral gene, *CpBV302*, was used as a control dsRNA (“dsCon”). **(A)** Effect of *Px-Duox* or *Px-Nox* RNAi on pathogenicity of *Bacillus thuringiensis kurstaki* (“BtK”) against L4 larvae of *P. xylostella*. RNAi-treated larvae were exposed to different BtK concentrations. Mortality was measured at 48 h post feeding. Each treatment was replicated three times. Each replication used 30 larvae. **(B)** Effect of BtK treatment on RNAi-treated larvae. BtK concentration was at 1.5 × 10^6^ spores/mL. Each treatment was replicated three times. A cytoskeletal gene, β*-actin*, was used as reference to normalize expression level. Asterisk indicates significant difference between control and treatment at Type I error = 0.05 (LSD test). “ns” represents no significant difference.

Under RNAi conditions of *Px-Duox* expression, BtK pathogenicity was significantly (*P* < 0.05) increased at all BtK concentrations (Figure 7A). BtK treatment up-regulated *Px-Duox* expression. However, it did not increase the gene expression under the RNAi conditions (Figure 7B). Similarly, RNAi specific to *Px-Nox* expression increased BtK pathogenicity, in which RNAi treatment prevented the up-regulation of *Px-Nox* expression in response to BtK treatment.

### Ptt Culture Broth Suppresses Expression Levels of *Px-Duox* and *Px-Nox*

**FIGURE 8:**
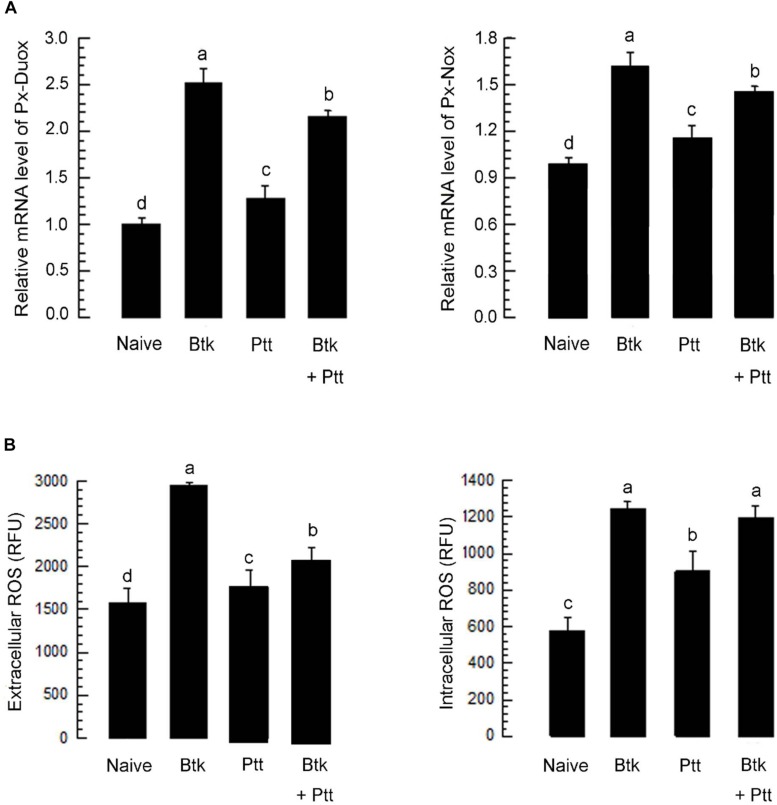
Suppressive effect of *P. temperata temperata* culture broth (“Ptt”) on ROS level up-regulated by *B. thuringiensis kurstaki* (BtK) in the gut of *P. xylostella*. BtK (1.5 × 10^6^ spores/mL)-treated cabbage leaves were fed to L4 larvae for 12 h by leaf-dipping method. Bacterial mixture treatment was prepared by suspending BtK cells (1.5 × 10^6^ spores/mL) in 100 mL of Ptt which was obtained when OD_600_ reached to 2.0. **(A)** Effect of Ptt on *Px-Duox* and *Px-Nox* expression in the gut. A cytoskeletal gene, β-actin, was used as a reference gene. **(B)** Effect of Ptt on ROS levels in gut lumen (“extracellular”) and gut epithelium (“intracellular”). All these expression analyses were replicated with three independent tissue preparations. Each replication used 15 insects. Different letters above standard deviation bars indicate significant differences among means at Type I error = 0.05 (LSD test).

**FIGURE 9:**
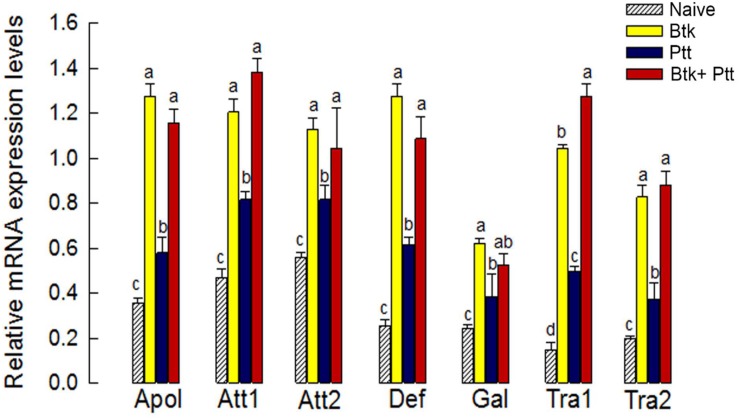
Influence of *P. temperata temperata* culture broth (“Ptt”) on antimicrobial peptide (AMP) genes up-regulated by *B. thuringiensis kurstaki* (BtK) in the gut of *P. xylostella*. BtK (1.5 × 10^6^ spores/mL)-treated cabbage leaves were fed to L4 larvae for 12 h by leaf-dipping method. Bacterial mixture treatment was prepared by suspending BtK cells (1.5 × 10^6^ spores/mL) in 100 mL of Ptt which was obtained when OD_600_ reached 2.0. Seven AMP genes analyzed in this study were: attacin 1 (Att1), attacin 2 (Att2), transferrin 1 (Tra1), transferrin 2 (Tra2), defensin (Def), apolipophorin III (Apol), and gallerimycin (Gal). Their expression levels in the gut of L4 larvae of *P. xylostella* were analyzed. β-Actin was used as an internal control. Each treatment was replicated three times with independent tissue preparations. Different letters above standard deviation bars in each AMP indicate significant differences among means at Type I error = 0.05 (LSD test).

The up-regulation of *Px-Duox* or *Px-Nox* expressions in response to BtK treatment was prevented by the addition of Ptt culture broth (Figure 8A). The inhibitory effect of Ptt culture broth was maintained at least for 30 h, leading to significant (*P* < 0.05) decrease of ROS levels in gut lumen (Supplementary Figure S1). Inhibitory effects of Ptt culture broth on the expression of these two oxidase genes were supported by decrease of ROS production levels after the mixture (BtK + Ptt broth) treatment (Figure 8B). However, Ptt culture broth did not suppress the up-regulation of AMP gene expression induced by BtK treatment (Figure 9).

### PLA_2_ Inhibitors Increases BtK Pathogenicity

**FIGURE 10:**
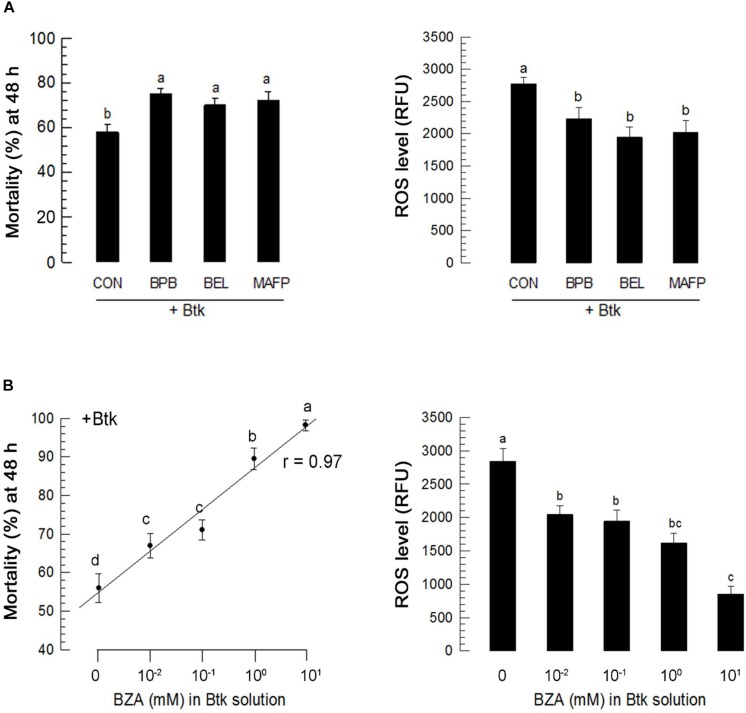
Influence of PLA_2_ inhibitors on *B. thuringiensis kurstaki* (BtK) toxicity against L4 larvae of *P. xylostella*. Three PLA_2_ inhibitors included 4-bromophenacyl bromide (BPB, sPLA_2_ inhibitor), bromoenol lactone (BEL, iPLA_2_ inhibitor), and methyl arachidonyl fluorophosphonate (MAFP, cPLA_2_ inhibitor). Each inhibitor was injected to the larval hemocoel at a concentration of 1 μg/larva. “CON” represents injection of solvent used to dissolve inhibitors. At 3 h post-injection of inhibitors, larvae were fed with BtK (1.5 × 10^6^ spores/mL) for 12 h. **(A)** Effects of different PLA_2_ inhibitors on pathogenicity of BtK against L4 larvae of *P. xylostella*. Each treatment was replicated three times. Each replication used 30 larvae. Mortality was measured at 48 h post feeding. **(B)** Effects of different PLA_2_ inhibitors on ROS level in the gut lumen. Each treatment was replicated three times. Each replication used 30 larvae. ROS levels were measured after BtK feeding experiment. Dose mortality of BZA against L4 larvae of *P. xylostella*. Different BZA concentrations were prepared in Btk (1.5 × 10^6^ spores/mL). Each treatment was replicated three times. Each replication used 30 larvae. Mortality was measured at 48 h post feeding. Regression coefficient (r) was measured between dose and mortality. Effects of different BZA concentrations on ROS level in the gut lumen of L4 larvae fed with BtK. At 0 mM, the same volume of solvent used to dissolve BZA was added to BtK suspension. Each treatment was replicated three times. Each replication used 15 larvae. ROS levels were measured at 12 h after feeding treatment. Different letters above standard deviation bars indicate significant differences among means at Type I error = 0.05 (LSD test).

Ptt culture broth contains PLA_2_ inhibitory factors (Seo et al., 2012). Its oral administration inhibited PLA_2_ activities in both hemolymph and fat body in the current study, suggesting that other commercial PLA_2_ inhibitors might increase BtK pathogenicity. To test this hypothesis, three different PLA_2_ inhibitors proven to be able to inhibit PLA_2_ activity in *Spodoptera exigua* (Sadekuzzaman and Kim, 2017) were used in this study for analysis. These three inhibitors significantly (*P* < 0.05) increased BtK pathogenicity (Figure 10A). BZA, a bacterial metabolite of Ptt (Seo et al., 2012), was used to test its efficacy on the increase of BtK pathogenicity (Figure 10B). BZA significantly (*P* < 0.05) increased BtK pathogenicity in a dose-dependent manner.

## Discussion

Two entomopathogenic bacterial genera, *Xenorhabdus* and *Photorhabdus*, can inhibit target insect immune responses by inhibiting eicosanoid biosynthesis through inhibition of PLA_2_ activity with their secondary metabolites (Kim et al., 2005). However, most analyses of immunosuppression have been focused on cellular and humoral immunity in hemocoel because bacteria can infect and multiply in the insect hemocoel (Kim et al., 2018a). Several studies have shown that Bt pathogenicity can be enhanced by the addition of bacterial culture broth or metabolite compounds (Jung and Kim, 2007; Kwon and Kim, 2008). Bt is pathogenic to target insects by oral infection. Its toxins are activated in the gut, in which host defense mechanisms operate to counteract the bacterial pathogenicity (Bravo et al., 2011). Thus, attacks derived from gut epithelium should be overcome to give realistic control efficacy. This study provides evidence that defense factor derived from gut epithelium can be prevented by adding culture broth of Ptt.

Bacterial culture broths of all 14 different *Xenorhabdus* and *Photorhabdus* strains enhanced BtK virulence. Especially, the culture broth of Ptt was superior in reducing BtK LC_50_ and increasing speed-to-kill of target insects. The Ptt bacterial culture broth also inhibited the activity of PLA_2_ in the hemolymph. All these observations support a hypothesis that *Xenorhabdus* and *Photorhabdus* can inhibit eicosanoid biosynthesis of target insects for their pathogenicity (Kim et al., 2005). Septicemia has been regarded as a main lethal pathogenicity of Bt (Broderick et al., 2006). Bt-induced septicemia has been well demonstrated in *Spodoptera littoralis*, in which RNAi-mediated silencing of an immune gene can reduce nodulation response and result in significant enhancement of host larval mortality with a remarkable predominance of *Serratia* and *Clostridium* species in the hemolymph (Caccia et al., 2016). Eicosanoids can mediate cellular and humoral immune responses to defend the host against fatal bacterial infection (Kim et al., 2018c). Inhibition of PLA_2_ catalytic activity by Ptt bacterial culture broth could prevent eicosanoid biosynthesis and lead to immunosuppression, which is a favorable condition for inducing septicemia. However, commensal bacterial infection is preceded by lesion of Bt against gut epithelium to allow the passage of bacteria. Thus, any damage of Bt or Bt toxin to the gut lumen would be unfavorable for Bt pathogenicity.

Reactive oxygen species was detected in both gut lumen or within epithelial cells of *P. xylostella* larvae. Administration of Ptt bacterial culture broth or an antioxidant (vitamin E) suppressed ROS levels in the gut, which resulted in an increase in bacterial growth within the gut lumen. Two ROS-producing enzymes (Px-Duox and Px-Nox) were predicted. Their gene expressions were detected in all developmental stages and tissues of *P. xylostella*. Both ROS-producing genes shared similar domain structures. Like Px-Nox, Px-Duox consists of a gp91phox homology domain and an EF-hand Ca^2+^-binding pocket. However, unlike Nox genes, an extracellular peroxidase homology domain (PHD) is found in Px-Duox, which is a distinct character of Duox (Bae et al., 2010). Mutant Duox with deletion of the PHD domain cannot function normally for host defense system (Ha et al., 2005). ROS synthesis begins with the intracellular domain. It is secreted from the extracellular domain. Briefly, the intracellular gp91phox homology domain can extract electrons from NADPH + H^+^. These electors are then delivered to two heme structures in transmembrane domain, subsequently forming superoxide and hydrogen peroxide. The extracellularly generated H_2_O_2_ is then subjected to peroxidation by extracellular PHD to generate microbicidal HOCl. In addition to microbicidal effects of Duox-dependent ROS, Duox is involved indirectly in epithelial cell renewal through the activation of intestinal stem cells during gut infection (Buchon et al., 2009). The Duox in zebrafish epidermal cells is involved in paracrine signaling to eliminate wound inflammation (Niethammer et al., 2009). It has an antimicrobial function in intestinal epithelium (Flores et al., 2010). In *Anopheles gambiae*, peroxidases and Duox contribute to the formation of a di-tyrosine network between the gut epithelial and peritrophic membrane, leading to decreased gut permeability to immune elicitors. Pathogen-specific immune responses of the host are triggered by disruption of this network (Kumar et al., 2010). Nox can mediate various oxidation reactions against xenobiotic compounds or organisms for defense in human neutrophils by producing ROS to kill microbial pathogens after phagocytosis (Henderson and Chappell, 1996). Like human neutrophils, insect hemocytes appear to have a similar Nox system to produce ROS for immune response (Slepneva et al., 1999; Bergin et al., 2005; Nappi and Christensen, 2005). In *S. exigua*, Nox is crucial for effective phagocytosis by increasing ROS levels in hemocytes. Its gene expression is induced by eicosanoids (Park et al., 2015). Our current analyses showed that RNAi specific to *Px-Duox* suppressed extracellular levels of ROS as well as intracellular ROS while RNAi specific to *Px-Nox* suppressed only intracellular ROS level. Ptt bacterial culture broth suppressed both ROS levels by inhibiting expression levels of *Px-Nox* and *Px-Duox*. It remains unknown how Ptt bacterial culture broth can suppress oxidase gene expression. In *Drosophila*, bacteria-originated uracil can up-regulate Duox activity via G protein-coupled receptor and activate Gαq-PLCβ-Ca^2+^ pathway to increase the enzymatic activity of Duox because Duox contains Ca^2+^-binding domain (Ha et al., 2009b). Gαq-PLCβ-Ca^2+^ pathway can also activate MEKK1-MKK3-p38 MAPK and up-regulate the expression level of Duox gene (Ha et al., 2009b). This suggests that there might be a cross-talk between Gαq-PLCβ-Ca^2+^ pathway and eicosanoid signaling. This needs to be explored in future studies.

In contrast, Ptt culture broth did not suppress the inducible AMP gene expression in response to BtK infection. BtK infection significantly induced expression levels of seven AMP genes. The addition of Ptt culture broth to BtK did not suppress the up-regulation of AMP genes. Bacterial homeostasis in *Drosophila* gut is under the control of Imd-AMP as well as Duox-ROS innate immune mechanisms (Kim and Lee, 2014). Eicosanoids are required for AMP expression via Imd pathway in *Drosophila* (Yajima et al., 2003). *P. xylostella* also possesses both Toll and Imd signal pathways from genome annotation (Xia et al., 2015). The current study showed that BtK infection significantly increased both sPLA_2_ and cPLA_2_ activities. PLA_2_ activities were inhibited by Ptt culture broth. The suppression of PLA_2_ activity may be directly induced by inhibitory binding of the bacterial secondary metabolites to the enzyme or indirectly induced by suppression of PLA_2_ gene expression. The latter hypothesis is now under testing in a subsequent study. On the other hand, the addition of Ptt culture broth to BtK infection failed to prevent the induction of AMP gene expression. This suggests that Imd pathway in the gut might be regulated by unique signal pathways that might not be identical to those in hemocytes and fat bodies.

Enhancement of BtK virulence by Ptt culture broth in inhibiting PLA_2_ activity proposed an application of other PLA_2_ inhibitors. All three different inhibitors specific to each of three types of PLA_2_s significantly enhanced BtK virulence. Moreover, BZA identified from Ptt culture broth (Seo et al., 2012) can also significantly enhance BtK virulence. All these observations support the practical application of Ptt culture broth to effectively control *P. xylostella* by mixing Bt insecticides in a form of Bt-Plus (Kim et al., 2015).

**FIGURE 11:**
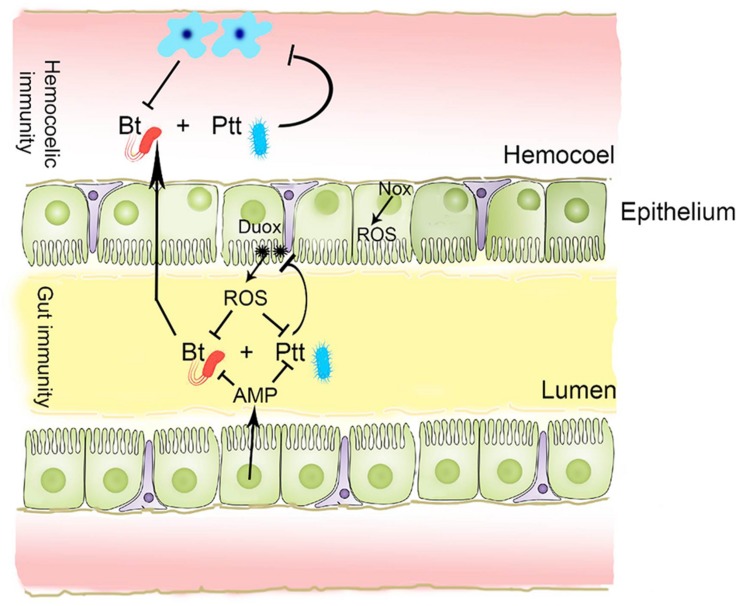
A schematic representation of gut immunity against *B. thuringiensis* (Bt) and its suppression by secondary metabolites of *P. temperata temperata* (Ptt). In response to Bt infection, gut epithelium releases antimicrobial peptide (AMP) and reactive oxygen species (ROS) into gut lumen. Both Duox and Nox are responsible for ROS synthesis. Ptt metabolites can inhibit *Duox* expression to prevent up-regulation of ROS induced by Bt infection.

These results suggest that BtK infection in *P. xylostella* can lead to up-regulation of ROS levels in the gut lumen as well as AMP gene expression in the gut epithelium (Figure 11). Secondary metabolites present in the Ptt culture broth can suppress gene expression of *Px-Duox* or *Px-Nox* to prevent ROS production via PLA_2_ inhibition to shutdown eicosanoid biosynthesis. The reduction of eicosanoid immune mediators could lead to immunosuppression, which then enhances BtK pathogenicity.

## Data Availability Statement

The datasets generated for this study can be found in the GenBank accession number: XP_011558844.1.

## Author Contributions

YK designed the work. SS performed the experiment. YK and SS wrote the manuscript.

## Conflict of Interest

The authors declare that the research was conducted in the absence of any commercial or financial relationships that could be construed as a potential conflict of interest.

## Footnotes

1 http://www.ncbi.nlm.nih.gov

2 https://www.ebi.ac.uk

3 http://pfam.xfam.org

## Supplementary Material

FIGURE S1 Response of *Px-Duox* and *Px-Nox* expressions in larval gut of *P. xylostella* during oral infection. **(A)** Expression levels of *Px-Duox* at different time points in whole guts after oral infection with BtK and Ptt mixture. **(B)** Expression levels of *Px-Nox* at different time points in whole guts after oral infection with BtK and Ptt mixture. **(C)** Intracellular and **(D)** extracellular intestinal ROS levels quantified at different time points after oral infection. The mixture treatment was prepared by suspending BtK cells (1.5 × 10^6^ spores/mL) in 100 mL of Ptt culture broth. Control used without any bacterial treatment (“No bac”).

TABLE S1 List of primers used in this study.

TABLE S2 Primer sequences of seven AMP genes assessed in this study.

TABLE S3 GenBank accession numbers used for phylogenetic analysis.
