# Supplementary material for: Dual Oxidase-Derived Reactive Oxygen Species Against Bacillus thuringiensis and Its Suppression by Eicosanoid Biosynthesis Inhibitors
Source: Front Microbiol. 2020 Mar 27;11:528. doi: 10.3389/fmicb.2020.00528 (PMC7120046; doi:10.3389/fmicb.2020.00528)
Supplement: FIGURE S1 — Response of Px-Duox and Px-Nox expressions in larval gut of P. xylostella during oral infection. (A) Expression levels of Px-Duox at different time points in whole guts after oral infection with BtK and Ptt mixture. (B) Expression levels of Px-Nox at different time points in whole guts after oral infection with BtK and Ptt mixture. (C) Intracellular and (D) extracellular intestinal ROS levels quantified at different time points after oral infection. The mixture treatment was prepared by suspending BtK cells (1.5 × 10^6^ spores/mL) in 100 mL of Ptt culture broth. Control used without any bacterial treatment (“No bac”). [file Data_Sheet_1.PDF]

Supplementary data

Table S1. List of primers used in this study

Primer	Uses	Sequence (5′ - 3′)	Annealing temperature (ºC)	Expected size of PCR product (bp)
Px-Duox	RT-PCR	GATGTCAACAGTGCCCATAGA<br>ATTGTCCAGTCGTCCATCATAC	52	310
Px-Nox	RT-PCR	GGTGAAGCTGAAACCCTACTT<br>CCGCGATCATAACACCAGTAA	52	311
Q_Px-Duox	RT-qPCR	AACGGACTCCCGGACTATAA<br>CGTCCATCATACGCCTGAATAA	52	137
Q_Px-Nox	RT-qPCR	GCAGCATCAAGTCTCACAAGA<br>CCATTGCACTCCTCCTCAATAC	52	91
T7_Px-Duox	dsRNA	TAATACGACTCACTATAGGGAGA<br>GATGTCAACAGTGCCCATAGA<br>TAATACGACTCACTATAGGGAGA<br>ATTGTCCAGTCGTCCATCATAC	52	310
T7_Px-Nox	dsRNA	TAATACGACTCACTATAGGGAGA<br>GGTGAAGCTGAAACCCTACTT<br>TAATACGACTCACTATAGGGAGA<br>CCGCGATCATAACACCAGTAA	52	311
dsCON	dsCon	CCCACTAGTGTCCTCATCACCTCC<br>TCAAAC<br>CCCAAGCTTCAGAGTCACCGTTG<br>CAAGTA	52	520
β-actin	RT-PCR and RT-qPCR	GCGACTTGACCGACTACCT<br>GCCGCAAGATTCCATACCC	52	272

* Underlined sequences represent T7 sequence

Table S2. Primer sequences of 7 AMP genes assessed in this study

Gene	Acronym	Sequence (5′ - 3′)	Annealing temperature (ºC)	Expected size (bp)
Apolipophorin III	Apol	AGTGTCGCCAAGTTGTTCGTG	52	420
		CTCCTGCGCGGTGTTCTGCA		
Attacin 1	Att1	GCTTTCCTCTCCAGGAATATG	52	276
		CCTTAGAGTAAATCCAGTGG		
Attacin 2	Att2	TCCCGAATGTGCCCAACTTC	52	254
		GAAAGATCTGCCGAAAGTAAG		
Defensin	Def	ATGGGTGTTAAGGTAATAAATGTG	52	303
		GCAACTACATGTATGACTAACGC		
Gallerimycin	Gal	TCAGTCATGAAAGCTTGCGTA	52	222
		TCGCACACATTGGCATCCATTC		
Transferrin 1	Tra1	GTCCCTCTCTGTCCTGAAGG	52	370
		CAGAAACACGAAGAAAGATGG		
Transferrin 2	Tra2	GATGTTCTGGCGCAGCTGTC	52	288
		CCGGCTGAACGCAAACACAG		

Table S3. GenBank accession numbers used for phylogenetic analysis

Species	Acronym	Gene	GenBank accession number
Aedes aegypti	A.aegDuox	Duox	XP_021700460.1
Agrilus planipennis	A.plaDuox	Duox	XP_018323658.1
Anopheles darlingi	A.darDuox	Duox	ETN58940.1
Anoplophora glabripennis	A.glaDuox	Duox	XP_018563303.1
Bactrocera dorsalis	B.dorDuox	Duox	AKS43593.1
Ceratitis capitata	C.capDuox	Duox	XP_004533990.1
Cryptotermes secundus	C.secDuox	Duox	PNF38268.1
Dendroctonus ponderosae	D.ponDuox	Duox	XP_019766183.1
Diabrotica virgifera	D.virDuox	Duox	XP_028130484.1
Drosophila persimilis	D.perDuox	Duox	XP_026843273.1
Formica exsecta	F.exsDuox	Duox	XP_029676591.1
Helicoverpa armigera	H.armDuox	Duox	XP_021185531.1
Leptinotarsa decemlineata	L.decDuox	Duox	XP_023012394.1
Lucilia cuprina	L.cupDuox	Duox	KNC33589.1
Onthophagus taurus	O.tauDuox	Duox	XP_022920959.1
Ostrinia furnacalis	O.furDuox	Duox	XP_028176585.1
Papilio machaon	P.macDuox	Duox	KPJ08497.1
Plutella xylostella	P.xylDuox, Px-Duox	Duox	XP_011558844.1
Solenopsis invicta	S.invDuox	Duox	XP_011156000.1
Spodoptera litura	S.litDuox	Duox	XP_022813998.1
Trichoplusia ni	T.niDuox	Duox	XP_026735041.1
Zeugodacus cucurbitae	Z.cucDuox	Duox	XP_011189298.1
Zootermopsis nevadensis	Z.nevDuox	Duox	XP_021932850.1
Bombyx mori	B.morNox	Nox	XP_012553051.1
Helicoverpa armigera	H.armNox	Nox	XP_021193767.1
Papilio machaon	P.macNox	Nox	XP_014368051.1
Spodoptera exigua	S.exiNox	Nox	AKM16733.1
Spodoptera litura	S.litNox	Nox	XP_022820068.1
Trichoplusia ni	T.niNox	Nox	XP_026742780.1
Pieris rapae	P.rapNox	Nox	XP_022118603.1
Plutella xylostella	P.xylNox, Px-Nox	Nox	XP_011562708.1

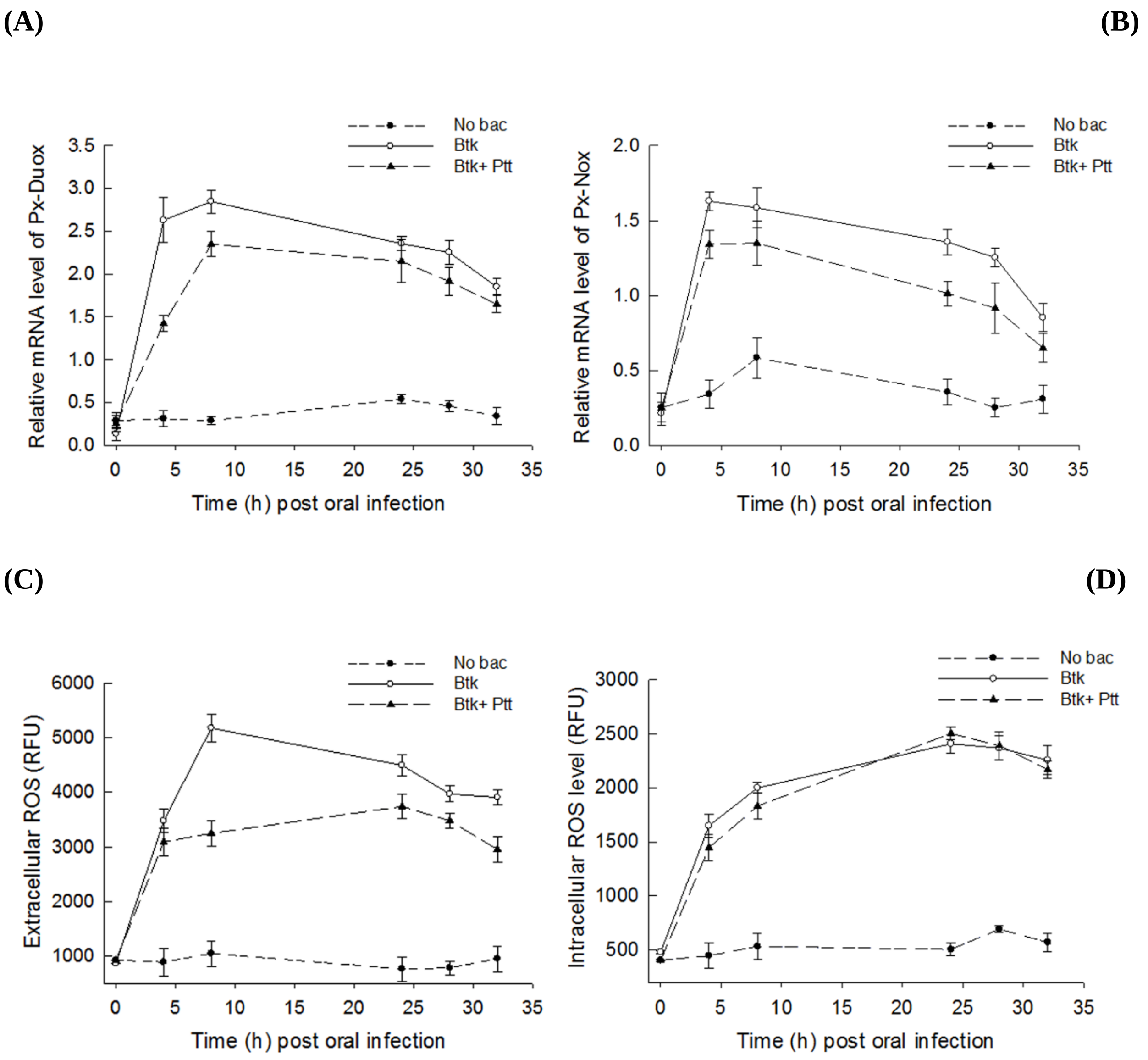
(A)
(B)
No bac
Btk
Btk+ Ptt
Relative mRNA level of Px-Duox
Time (h) post oral infection
Relative mRNA level of Px-Nox
Time (h) post oral infection
(C)
(D)
No bac
Btk
Btk+ Ptt
Extracellular ROS (RFU)
Time (h) post oral infection
Intracellular ROS level (RFU)
Time (h) post oral infection

Fig. S1
